# The effect of immunosuppressive therapies on the endothelial host response in critically ill COVID-19 patients

**DOI:** 10.1038/s41598-024-59385-w

**Published:** 2024-04-20

**Authors:** M. A. Slim, E. H. T. Lim, L. A. van Vught, A. M. Tuip-de Boer, E. Rademaker, J. L. G. Haitsma Mulier, J. J. Engel, The Amsterdam UMC COVID-19 Biobank Study Group, M. van Agtmael, A. G. Algera, B. Appelman, F. Baarle, M. Beudel, H. J. Bogaard, M. Bomers, L. D. Bos, M. Botta, J. de Brabander, G. de Bree, S. de Bruin, M. Bugiani, E. Bulle, D. T. P. Buis, O. Chouchane, A. Cloherty, M. C. F. J. de Rotte, M. Dijkstra, D. A. Dongelmans, R. W. G. Dujardin, P. Elbers, L. Fleuren, S. Geerlings, T. Geijtenbeek, A. Girbes, B. Goorhuis, M. P. Grobusch, L. Hagens, J. Hamann, V. Harris, R. Hemke, S. M. Hermans, L. Heunks, M. Hollmann, J. Horn, J. W. Hovius, M. D. de Jong, R. Koning, E. H. T. Lim, N. van Mourik, J. Nellen, E. J. Nossent, F. Paulus, E. Peters, D. A. I. Piña-Fuentes, T. van der Poll, B. Preckel, J. Raasveld, T. Reijnders, M. Schinkel, M. J. Schultz, F. A. P. Schrauwen, A. Schuurman, J. Schuurmans, K. Sigaloff, M. A. Slim, P. Smeele, M. Smit, C. S. Stijnis, W. Stilma, C. Teunissen, P. Thoral, A. M. Tsonas, P. R. Tuinman, M. van der Valk, D. Veelo, C. Volleman, H. de Vries, L. A. Vught, M. van Vugt, D. Wouters, A. H. Zwinderman, M. C. Brouwer, W. J. Wiersinga, A. P. J. Vlaar, D. van de Beek, The Radboudumc Center for Infectious Diseases COVID-19 Study Group, Anneke Hijmans, Bram van Cranenbroek, Chantal Bleeker-Rovers, Cor Jacobs, Esther Fasse, Esther van Rijssen, Esther Taks, Fieke Weren, Gerine Nijman, Hans Koenen, Heidi Lemmers, Heiman Wertheim, Helga Dijkstra, Hetty van der Eng, Hidde Heesakkers, Ilse Kouijzer, Irma Joosten, Jaap ten Oever, Jacobien Hoogerwerf, Janette Rahamat-Langendoen, Jelle Gerretsen, Jeroen Schouten, Joost Hopman, Josephine van de Maat, Kiki Schraa, Leonie Buijsse, Liesbeth van Emst, Liz Fransman, Manon Kolkman, Margreet Klop-Riehl, Martin Jaeger, Nicole Waalders, Niklas Bruse, Noortje Rovers, Pleun Hemelaar, Priya Debisarun, Quirijn de Mast, Reinout van Crevel, Remi Beunders, Ruben Smeets, Simone Moorlag, Sjef van der Velde, Tim Frenzel, Tirsa van Schaik, Trees Jansen, Wout Claassen, P. Pickkers, F. L. van de Veerdonk, A. P. J. Vlaar, L. P. G. Derde, N. P. Juffermans

**Affiliations:** 1 Center for Experimental and Molecular Medicine, Amsterdam University Medical Centers, University of Amsterdam, Amsterdam, The Netherlands; 2 Department of Intensive Care, Amsterdam University Medical Centers, University of Amsterdam, Amsterdam, The Netherlands; 3 Laboratory of Experimental Intensive Care and Anesthesiology, Amsterdam University Medical Centers – Location Academic Medical Center, University of Amsterdam, Amsterdam, The Netherlands; 4 Department of Intensive Care Medicine, University Medical Center Utrecht, Utrecht, The Netherlands; 5 Julius Centre for Health Sciences and Primary Care, University Medical Center Utrecht, Utrecht, The Netherlands; 6 Department of Internal Medicine, Radboud University Medical Center, Nijmegen, The Netherlands; 7 Amsterdam University Medical Centers, University of Amsterdam, Amsterdam, The Netherlands; 8 Center for Infectious Diseases, Radboud University Medical Center, Nijmegen, The Netherlands; 9 Department of Intensive Care Medicine, Radboud University Medical Center, Nijmegen, The Netherlands; 10 Department of Intensive Care Medicine, Erasmus Medical Center, Rotterdam, The Netherlands; 11 Department of Intensive Care, Amsterdam University Medical Center, Meibergdreef 9, Room G3-220, 1105 AZ Amsterdam, the Netherlands; 12 Department of Infectious Diseases, Amsterdam University Medical Centers, University of Amsterdam, Amsterdam, The Netherlands; 13 Department of Neurology, Amsterdam University Medical Centers, University of Amsterdam, Amsterdam, The Netherlands; 14 Department of Pulmonology, Amsterdam University Medical Centers, University of Amsterdam, Amsterdam, The Netherlands; 15 Department of Pathology, Amsterdam University Medical Centers, University of Amsterdam, Amsterdam, The Netherlands; 16 Department of Experimental Immunology, Amsterdam University Medical Centers, University of Amsterdam, Amsterdam, The Netherlands; 17 Department of Clinical Chemistry, Amsterdam University Medical Centers, University of Amsterdam, Amsterdam, The Netherlands; 18 Amsterdam UMC Biobank Core Facility, Amsterdam University Medical Centers, University of Amsterdam, Amsterdam, The Netherlands; 19 Department of Radiology, Amsterdam University Medical Centers, University of Amsterdam, Amsterdam, The Netherlands; 20 Department of Anesthesiology, Amsterdam University Medical Centers, University of Amsterdam, Amsterdam, The Netherlands; 21 Department of Medical Microbiology, Amsterdam University Medical Centers, University of Amsterdam, Amsterdam, The Netherlands; 22 Neurochemical Laboratory, Amsterdam University Medical Centers, University of Amsterdam, Amsterdam, The Netherlands; 23 Department of Clinical Epidemiology, Biostatistics and Bioinformatics, Amsterdam University Medical Centers, University of Amsterdam, Amsterdam, The Netherlands

**Keywords:** COVID-19, Host response, Immunomodulatory treatment, Complement, Coagulation, Endothelial function, Biomarkers, Viral infection, Immunosuppression

## Abstract

While several effective therapies for critically ill patients with COVID-19 have been identified in large, well-conducted trials, the mechanisms underlying these therapies have not been investigated in depth. Our aim is to investigate the association between various immunosuppressive therapies (corticosteroids, tocilizumab and anakinra) and the change in endothelial host response over time in critically ill COVID-19 patients. We conducted a pre-specified multicenter post-hoc analysis in a Dutch cohort of COVID-19 patients admitted to the ICU between March 2020 and September 2021 due to hypoxemic respiratory failure. A panel of 18 immune response biomarkers in the complement, coagulation and endothelial function domains were measured using ELISA or Luminex. Biomarkers were measured on day 0–1, day 2–4 and day 6–8 after start of COVID-19 treatment. Patients were categorized into four treatment groups: no immunomodulatory treatment, corticosteroids, anakinra plus corticosteroids, or tocilizumab plus corticosteroids. The association between treatment group and the change in concentrations of biomarkers was estimated with linear mixed-effects models, using no immunomodulatory treatment as reference group. 109 patients with a median age of 62 years [IQR 54–70] of whom 72% (n = 78) was male, were included in this analysis. Both anakinra plus corticosteroids (n = 22) and tocilizumab plus corticosteroids (n = 38) were associated with an increase in angiopoietin-1 compared to no immune modulator (n = 23) (beta of 0.033 [0.002–0.064] and 0.041 [0.013–0.070] per day, respectively). These treatments, as well as corticosteroids alone (n = 26), were further associated with a decrease in the ratio of angiopoietin-2/angiopoietin-1 (beta of 0.071 [0.034–0.107], 0.060 [0.030–0.091] and 0.043 [0.001–0.085] per day, respectively). Anakinra plus corticosteroids and tocilizumab plus corticosteroids were associated with a decrease in concentrations of complement complex 5b-9 compared to no immunomodulatory treatment (0.038 [0.006–0.071] and 0.023 [0.000–0.047], respectively). Currently established treatments for critically ill COVID-19 patients are associated with a change in biomarkers of the angiopoietin and complement pathways, possibly indicating a role for stability of the endothelium. These results increase the understanding of the mechanisms of interventions and are possibly useful for stratification of patients with other inflammatory conditions which may potentially benefit from these treatments.

## Introduction

Mortality in patients with coronavirus disease 2019 (COVID-19) related acute respiratory distress syndrome (ARDS) remains high^1^, even though the introduction of the Omicron variant of COVID-19, vaccination, improved treatment, and immunity acquired from previous infections, have significantly reduced critical illness from the disease^2–4^. Immune dysregulation is an important component of the pathophysiology of COVID-19 related ARDS^5^. Clinical trials have shown improved outcomes for corticosteroids, interleukin (IL)6-receptor antagonists and baricitinib in critically ill patients^6^. In addition, anakinra, an IL-1 receptor antagonist, reduced mortality only in hospitalized patients with high baseline concentration of soluble urokinase plasminogen activator receptor^7^.

Immune dysregulation may contribute to the development of thrombotic complications, especially in critically ill patients with COVID-19, with an incidence as high as 34%^8,9^. High plasma concentrations of fibrinogen, von Willebrand factor (vWF) and D-dimer measured in these patients, suggest presence of a COVID-19-associated coagulopathy^10,11^. Additionally, the endothelium is activated by severe acute respiratory syndrome coronavirus 2 (SARS-CoV-2), leading to cellular damage^12^, resulting in a pro-thrombotic phenotype. This endotheliopathy further leads to elevated concentrations of plasminogen activator inhibitor 1 (PAI-1), vWF and increased platelet activation. Taken together, these changes may result in venous, arterial, and microvascular thrombosis^9^.

Complement activation is also part of the pathogenesis of COVID-19^13^ and is associated with poor outcomes^14^. The complement and coagulation pathways are closely linked^15,16^. Complement 3 (C3) and activated Complement 5 (C5) contribute to enhanced expression of tissue factor on endothelial cells and leukocytes, and both mediators recruit leukocytes and enhance cytokine release after the initiation of the immune response^9,17,18^. This host immune response leads to additional endothelial cell damage further contributing to a pro-thrombotic state in COVID-19 patients^17,19^.

Beneficial findings of immunosuppressive therapies in COVID-19 related ARDS may stimulate further investigation into interventions in ARDS due to other causes, or in other syndromes of critical illness. Though randomized clinical trials (RCTs) are important to find effective therapies for patients, heterogeneity of treatment effects in subpopulations is likely^20^. These effects are difficult to prove, but have been suggested to play a role in previous critical care RCTs^21–26^. Thus, there is a need to understand the underlying biological changes in patients, to identify the subgroup of patients most likely to benefit. As such, it is important to understand the (specific) effects of immunosuppressive therapies on endothelial host immune response in critically ill patients with COVID-19. Our aim was to investigate the association between various immunosuppressive therapies (corticosteroids, tocilizumab or anakinra) and the change in concentrations of complement- and endothelial activation pathways in these patients.

## Methods

### Study design and population

The study cohort consisted of prospectively collected clinical data and samples from a cohort of Dutch critically ill patients with COVID-19. This cohort included data and samples from three Dutch academic hospitals: the Amsterdam University Medical Centers (Amsterdam UMC), the University Medical Center Utrecht (UMCU), and the Radboud University Medical Center Nijmegen (Radboudumc). Informed consent was provided by the patient, or their legal representative if they were incapacitated, as per national legislation and local procedures. The relevant (biobanking) ethics committees of all three hospitals approved the study and all research was performed in accordance with relevant guidelines and regulations (approval for the Amsterdam UMC was granted by the biobank ethics committee 2020.182, for UMCU by the TCBio (protocol number 22-483), and for the Radboudumc by the local ethics committee CMO 2020 6344).

Adult patients were eligible for the study if they were admitted to the Intensive Care Unit (ICU) with COVID-19 as the main admission diagnosis (defined as clinical diagnosis of COVID-19 and/or a SARS-CoV-2-positive Polymerase chain reaction (PCR) from nasopharyngeal or tracheal swab on admission), received immunomodulatory treatment or standard of care (defined as no immunemodulatory treatment) and at least one ethylenediaminetetraacetic acid (EDTA) sample was obtained within 24 h after start of immunomodulatory treatment. For patients who did not receive immunomodulatory treatment, they were eligible if sampled on day 0 on 1 of ICU admission. Exclusion criteria were organ or bone marrow transplantation, human immunodeficiency virus Infection with CD4 cell count < 200 cells/μL and systemic immunosuppression prior to hospitalization (including systemic corticosteroids). Three groups of immunomodulatory treatment were defined: (1) corticosteroids alone, (2) corticosteroids combined with anakinra, and (3) tocilizumab combined with corticosteroids. The differences in treatment regimens per center can be found in the Supplementary methods. Subsequent samples taken from these patients were included to evaluate the development of biomarkers during the first week after treatment to investigate the association between various immunosuppressive therapies (corticosteroids, tocilizumab or anakinra) and the early change in concentrations of complement- and endothelial activation pathways in these patients since the immunosuppressive therapies were mostly administered or initiated upon ICU admission. Furthermore, looking at samples the first week after start of treatment would minimize the rate of missing samples. We grouped samples taken at day 2–4 (as day 3) and those taken at day 6–8 (as day 7).

### Sample size

The number of samples used is based on the number of available samples in the three Dutch biobanks combined; therefore, sample size calculation was not performed.

### Data collection

Clinical data were collected as part of clinical care (Radboudumc, Amsterdam UMC, UMCU) and as part of an intervention study investigating the best treatment for critically ill patients with COVID-19 (Randomized Embedded Multifactorial Adaptive Platform Trial for Community Acquired Pneumonia (REMAP-CAP)^27^; UMCU, Radboudumc). Collected clinical data included immunomodulatory treatment at ICU admission, patient demographics, Sequential Organ Failure Assessment (SOFA) and Acute Physiology and Chronic Health Evaluation (APACHE) II score, vital signs and laboratory test results at admission, as well as thrombotic events, length of ICU and hospital stay, duration of invasive mechanical ventilation and ICU- and in-hospital mortality. Ventilator-free days at 28 days were calculated as the number of days successfully liberated from mechanical ventilation, where for patients ventilated 28 days or more, or who died within 28 days, ventilator-free days were zero^28^.

### Biomarker assays

Serum was separated from whole blood samples, and stored hours at − 80 °C until assay. Biomarkers were measured using a Luminex platform or enzyme-linked immune sorbent assay (ELISA). A disintegrin and metalloproteinase with a thrombospondin type 1 motif, member 13 (ADAMTS13), angiopoietin-1 (ang-1), angiopoietin-2 (ang-2), complement 5a (C5a), D-dimer, ferritin, thrombomodulin, tissue factor, von Willebrand factor (vWF), syndecan-1, E-Selectin, P-selectin and PAI-1 were measured using Luminex multiplex assay (R&D Systems Inc., Minneapolis, United States), using the Bio-Plex 200 System (Bio-Rad Laboratories Inc., California, United States) in one batch (for more details and the detection limits see Supplementary Table 1). Fibrinogen, tissue Plasminogen Activator (tPA), complement 3a (C3a), complement complex 5b-9 (C5b-9), and Mannan-binding lectin serine protease 2 (MASP2) were measured using ELISA. Samples were randomly assigned to a plate before the analyses, which were executed according to the instruction of the manufacturer. Values below or above the detection limit that could not be extrapolated based on the standard curve were not included in the analyses (Supplementary Table 2). In order to obtain reference biomarker values, we analyzed samples of 15 healthy controls (age 20–30 years).

### Statistical analyses

Patients not treated with immunomodulatory treatments were used as the control group. Distribution of baseline characteristics and clinical outcomes was analyzed using histogram plots. Differences between treatment groups at baseline were tested with a Kruskal Wallis test as appropriate, and presented as median and interquartile ranges. Differences between treatment groups at baseline were tested with one-way ANOVA or a Kruskal Wallis test as appropriate, and presented as means and standard deviation; and median and interquartile ranges. All biomarkers were log10 transformed to better approach a normal distribution. The association of the concentration of biomarkers with treatment group was estimated using linear mixed-effects models (with the *lme4* package in R studio)^29^. Treatment group, sample day, and their interaction were included as fixed effects. Random intercepts were assigned to each subject. The effect of the treatment over time was determined by evaluating the interaction term and the 95% confidence interval (95% CI) of this term was calculated. Since this is an explorative study design, no formal sample size calculation was performed. The number of samples used was pragmatically based on the number of available samples in the three Dutch biobanks combined. All analyses were performed using R studio version 4.0.3 built under R. A p value of < 0.05 was considered statistically significant.

### Ethics declaration

Informed consent was provided by the patient, or their legal representative if they were incapacitated, as per national legislation and local procedures. The relevant (biobanking) ethics committees of all three hospitals (Amsterdam University Medical Centers, University Medical Center Utrecht, and Radboud University Medical Center Nijmegen) approved the study.

## Results

Two or more blood samples were available from 109 patients that fulfilled eligibility criteria. Patients were admitted between March 2020 and September 2021. Treatment groups were evenly distributed, with 21% of patients (n = 23) receiving no immunomodulatory treatment, 24% (n = 26) receiving corticosteroids only, 20% receiving (n = 22) anakinra (of which 21 also received corticosteroids), and 35% (n = 38) receiving tocilizumab plus corticosteroids. Corticosteroids administered were either dexamethasone (6 mg per day) or hydrocortisone (100 to 400 mg per day). The median [IQR] age of all patients was 62 [54–70] years, and 72% (n = 78) were males. Baseline variables did not significantly differ between the treatment groups (Table 1). The overall median [IQR] number of days from start of symptoms to start of immunomodulatory treatment or ICU admission (in case of no treatment) was 10 [7–14]. This was significantly higher for those who received anakinra compared to the other treatment groups (*p* < 0.001). The median [IQR] number of days between ICU admission and start of treatment was 0 [0–1] for corticosteroids, 0 [0–1] for tocilizumab, and 10 [0–13] for anakinra. This was because anakinra was either administered on admission (< 2 days after ICU admission, n = 9) or on clinical indication (median [IQR] day 13 [11–16 days], n = 13). The rate of pulmonary embolism was 35% (n = 38) and in-hospital mortality was 20% (n = 22), with no differences between treatment groups (*p* = 0.943 and *p* = 0.484). Baseline samples were available for all patients, samples on day 3 were available in 107 (98%) of patients and in 95 patients on day 7 (87%). No samples were missing related to early deaths, the shortest time between baseline and death was 6 days. Missing biomarker concentrations were not imputated. We performed quality assessment of the measured biomarkers (Supplementary Table 2).

**Table 1:** Baseline characteristics and clinical trajectory of patients with COVID-19 related ARDS.

	All patients n = 109	No immuno-modulation n = 23	Corticosteroids only n = 26	Anakinra + costicosteroids n = 22	Tocilizumab + corticosteroids n = 38	p value
Admission date		Mar–Apr 20	Mar 20–May 21	Mar–Nov 20	Nov 20–Sept 21	
Demographic characteristics						
Sex (male, no. (%))	78 (71.6)	16 (69.6)	19 (73.1)	17 (77.3)	26 (68.4)	0.894
Age, years (median [IQR])	62 [54, 70]	63 [53, 71]	62 [57, 67]	60 [54, 71]	63 [51, 72]	0.950
BMI, kg/m^3^ (median [IQR])	27.6 [24.8, 30.8]	25.8 [22.6, 29.2]	27.7 [24.7, 30.0]	26.7 [24.4, 28.5]	28.9 [26.0, 34.1]	0.022
Baseline characteristics						
SOFA score (median [IQR])	5 [3, 7]	5 [4, 7]	6 [4, 8]	6 [3, 7]	5 [3, 7]	0.364
APACHE II score (median [IQR])	13 [10, 17]	10 [10, 13]	13 [12, 16]	14 [12, 17]	13 [11, 18]	0.148
Diabetes (no. (%))	36 (33.0)	7 (30.4)	10 (38.5)	7 (31.8)	12 (31.6)	0.926
Immune deficiency* (no. (%))	6 (5.5)	0 (0.0)	2 (7.7)	2 (9.1)	2 (5.3)	0.546
Days since symptoms** (median [IQR])	10 [7, 14]	8 [7, 14]	9 [6, 13]	19 [13, 24]	9 [8, 12]	< 0.001
Laboratory values at baseline						
WBC (median [IQR])	9.8 [7.3, 13.5]	9.4 [6.9, 10.4]	8.7 [6.1, 10.6]	12.3 [10.4, 15.2]	8.8 [7.0, 13.1]	0.014
Platelets (median [IQR])	279 [203, 354]	269 [201, 360]	222 [173, 291]	368 [305, 482]	282 [220, 332]	0.002
CRP (median [IQR])	136 [88, 238]	181 [101, 237]	136 [100, 259]	113 [64, 232]	136 [83, 213]	0.837
Creatinine (median [IQR])	77 [60, 99]	65 [61, 93]	82 [67, 119]	85 [68, 108]	72 [59, 96]	0.331
Clinical outcomes						
Pulmonary thrombosis (no. (%))	38 (34.9)	9 (39.1)	9 (34.6)	8 (36.4)	12 (31.6)	0.943
Mechanical ventilation (no. (%))	90 (82.6)	22 (95.7)	19 (73.1)	21 (95.5)	28 (73.7)	0.029
Ventilator free days, days (median [IQR])	12 [0, 21]	13 [1, 20]	0 [0, 13]	3 [0, 14]	19 [2, 23]	0.112
Length of ICU stay, days (median [IQR])	13 [8, 26]	12 [9, 17]	18 [7, 39]	20 [13, 30]	10 [7, 16]	0.087
Length of hospital stay, days (median [IQR])	21 [13, 36]	18 [13, 26]	28 [14, 42]	28 [20, 45]	18 [12, 30]	0.014
ICU-hospital mortality (no. (%))	20 (18.3)	5 (21.7)	7 (26.9)	4 (18.2)	4 (10.5)	0.391
In-hospital mortality (no. (%))	22 (20.2)	6 (26.1)	7 (26.9)	4 (18.2)	5 (13.2)	0.484

*APACHE* Acute Physiology and Chronic Health Evaluation, *BMI* Body-mass index, *CRP* C-reactive protein, *ICU* intensive care unit, *SOFA* sequential organ failure assessment, *WBC* white blood count.

*Chemotherapy or radiation within 4 weeks of admission, high-dose steroid treatment (> 1.5 mg/kg methyl prednisolone or equivalent for ≥ 5 days), long-term treatment steroid treatment (> 20 mg/day of a steroid), other immunosuppressive treatment (methotrexate, rituximab, etc.), AIDS, acute leukemia, lymphoma, metastatic cancer, myeloma or other immunosuppressive disease.

**Since ICU-admission.

### Biomarkers at baseline

We found lower concentrations of vWF (6224.4 pg/ml vs. 9631.5 pg/ml, *p* = 0.005), E-selectin (16,342 pg/ml vs. 23,363 pg/ml, *p* < 0.001) and P-selectin (13,515 pg/ml vs. 19,817 pg/ml, *p* = 0.002) in patients receiving corticosteroids when compared to untreated patients at baseline. Those receiving anakinra had lower concentrations of ADAMTS-13 (486,643 pg/ml vs. 682,613 pg/ml, *p* = 0.005) and vWF (5674.5 pg/ml vs. 9631.5 pg/ml, *p* = 0.006) compared to untreated patients at baseline. Concentrations of C5b-9 (495,438 pg/ml vs. 675,505 pg/ml, *p* = 0.004), vWF (6271.0 pg/ml vs. 9631.5 pg/ml, *p* = 0.01), ang-2 (2739.9 pg/ml vs. 4479.9 pg/ml, *p* = 0.01) and P-selectin were lower (16,258 pg/ml vs. 19,817 pg/ml, *p* = 0.03) and tissue factor (84.5 pg/ml vs. 65.3 pg/ml, *p* = 0.027) was higher in patients receiving tocilizumab when compared to untreated patients. The concentrations of other biomarkers did not differ at baseline between untreated patients and those receiving corticosteroids, anakinra or tocilizumab (Supplementary Fig. 1). At baseline, we also compared biomarkers in patients with pulmonary embolism to patients without pulmonary embolism. Except for the concentration of PAI-1 (1,807,949 pg/ml vs. 1,011,185 pg/ml, *p* = 0.049), no differences were found (Supplementary Table 3). The biomarker concentrations on day 3 and 7 can are described in Supplementary Fig. 1.

### Effect of various treatments on biomarkers

**Figure 1:**
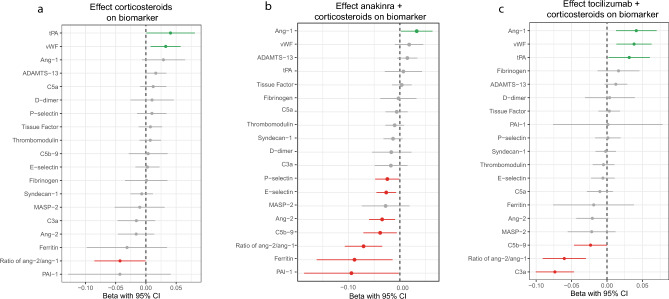
The effect of various treatments on the biomarker concentrations over time. The effect of various treatments (**a**) corticosteroids, (**b**) anakinra plus corticosteroids and (**c**) tocilizumab plus corticosteroids, on the biomarker concentrations over the first 7 days of treatment (or ICU-admission in case of no immunomodulation) when compared to no immunomodulatory treatments. *ADAMTS13* a disintegrin and metalloproteinase with a thrombospondin type 1 motif, member 13, *Ang-1* angiopoeitin-1, *Ang-2* angiopoeitin-2, *C3a* complement 3a, *C5a* complement 5a, *C5b-9* complement complex 5b-9, *MASP-2* Mannan-binding lectin serine protease 2, *PAI-1* plasminogen activator inhibitor-1, *tPA* tissue plasminogen activator, *vWF* von Willebrand factor.

In the linear mixed-effect models, treatment with corticosteroids was associated with a decrease in the ratio of ang-2/ang-1 and an increase in the concentrations of tPA and vWF over time (Fig. 1, Supplementary Table 4). The courses of other biomarkers were not affected.

Treatment with anakinra plus corticosteroids was associated with a decrease in C5b-9, ferritin, PAI-1, ang-2, ratio of ang-2/ang-1, E-selectin, and P-selectin over time (Fig. 1, Supplementary Table 4). On the other hand, it was associated with an increasing concentration of ang-1 over time. The courses of other biomarkers were not affected by treatment with anakinra plus corticosteroids.

Treatment with tocilizumab plus corticosteroids was associated with decreased concentrations of C3a, C5b-9 and the ratio of ang-2/ang-1 over time. We found an increased concentration of tPA, vWF and ang-1 (Fig. 1, Supplementary Table 4) during treatment. The concentration courses of other biomarkers were not associated with the treatment of tocilizumab plus corticosteroids.

## Discussion

In this study, we aimed to investigate the association of various immunosuppressive therapies (corticosteroids alone, anakinra plus corticosteroids, and tocilizumab plus corticosteroids) and the trajectories of biomarkers of endothelial host response over time, in critically ill patients with COVID-19. In our study, all immunomodulatory treatments for critically ill COVID-19 patients are associated with a decrease in the ratio of ang-2/ang-1 and in concentrations of C5b-9. These results underline the important roles for the angiopoietin and complement pathways in in the pathogenesis of COVID-19.

The most consistent impact of the various immunomodulatory treatments in this analysis is on the angiopoietin pathway, with a stronger impact of combination treatment then steroids alone. A decrease in the ratio of ang-2/ang-1 and increase in concentrations of ang-1, suggests a critical role for apoptosis via the endothelial tyrosine kinase receptor (Tie)-2. These two angiopoietins have an agonist/antagonist working mechanism on Tie-2, thereby influencing the integrity of the endothelium. Ang-1 stabilizes the endothelium, while ang-2 is pro-inflammatory and promotes endothelial cell apoptosis^30^. In ARDS due to other causes, higher ang-2 has been linked to the underlying pathogenesis of ARDS^31^. Ang-2 is normally downregulated by angiotensin-converting enzyme 2 (ACE2)^32^. Given that ACE2 is a receptor for SARS-CoV-2 entry in the host cells, leading to downregulation of ACE2 with ensuing endothelial dysfunction^33^, the ratio of ang-2/ang-1 is of particular relevance in COVID-19 induced ARDS. It has been previously described that treatment with dexamethasone decreases ang-2^34^. We extend these findings by showing that steroids combined with IL-1 or IL-6 receptor antagonists is associated with an amplified increase in ang-1 and steroids combined with IL-1 receptor antagonist is associated with a more pronounced decrease in ang-2 than steroids alone.

Our results suggest that complement inhibition seems to be another important pathway in immunomodulation by IL-1 and IL-6 receptor antagonists. In both treatment arms with anakinra plus corticosteroids and tocilizumab plus corticosteroids, a reduction in C5b-9 complement concentrations was seen. Similarly, clinical studies in rheumatoid arthritis patients showed a reduction in complement concentrations after the use of tocilizumab (without corticosteroids)^35^. This is in line with previous findings, suggesting IL-6 induces complement components such as C3 in the liver and C5a receptor in solid organs^36^. In previous studies, treatment with anakinra was associated with a reduction in complement concentrations in mice with myasthenia gravis^37^ and in patients with systemic lupus erythematosus^38^. It may however be more efficient to target complement directly instead of indirectly via IL-1 modulation. In line, studies in COVID-19 related ARDS show blocking complement activation in severe patients is associated with improved outcomes^39,40^.

C5a, a potent anaphylatoxin that plays a key role in severe COVID-19^18,41,42^, was only decreased on day 3 in our study, in patients receiving tocilizumab plus corticosteroids. Previous research has shown that blockade of C5a requires a specifically targeted inhibition as C5a can be generated outside the common complement pathways through direct enzymatic cleavage by trypsin and thrombin^42–44^. A recent phase 3 study investigating the potential effect of direct C5a inhibition in patients with critically ill COVID-19 showed a 23% relative mortality reduction^18^. In that study, only a minority of patients received tocilizumab concurrently. Whether adding complement inhibitors to treatment with tocilizumab plus corticosteroids can improve outcomes for critically ill patients with COVID-19 is unknown.

Anakinra was associated with a reduction in ferritin, which concurs with the macrophage activation syndrome which may be present in severely ill COVID-19 patients^7^. This effect was not observed with tocilizumab (plus corticosteroid) treatment. Also, IL-1 blockade led to greater reduction of E-selectin, P-selectin and PAI-1 compared to IL-6 blockade, suggesting enhanced endothelial ‘stabilization’^45^. It can be hypothesized that combined therapy with both treatments could further improve outcomes for these patients. A positive result of double-therapy of both IL-1 and IL-6 blockade, or even triple therapy with dexamethasone in 61% of the patients, was observed in a small case series of 31 hospitalized patient with moderate to severe COVID-19 were 81% was discharged^46^, but it has not yet been established in a randomized clinical trial whether simultaneous treatment with both IL-1 and IL-6 blockade might have an advantage over therapy with either one of these compounds.

Contrary to our expectations, treatment with corticosteroids alone as well as combination therapy with tocilizumab and corticosteroids was associated with increased concentrations of vWF and tPA, suggesting enhanced tissue injury and endothelial activation. Of note, there was no difference in the incidence of pulmonary embolisms between the two treatment groups. We hypothesize that the significantly elevated concentrations of PAI-1 from lung epithelium and endothelial cells contributing to a hypofibrinolytic state are counterbalanced by tPA-induced impairment of fibrinolysis, with no net effect on thrombotic risk^47^ in our study. It remains unclear why these treatments resulted in increased concentration of vWF and tPA. Treatment with corticosteroids has been described to increase concentrations of vWF in healthy volunteers^48,49^, by activating endothelial cells and leukocytes, as shown by increased haemostatic gene expression favoring pro-adhesiveness and neutrophil adhesion^50^. In high-risk cardiovascular patients, tocilizumab was demonstrated to improve endothelial function (assessed by flow-mediated dilation)^51^. In addition, dexamethasone has been suggested to improve COVID-19-related endothelial injury^34^.

Our study has several limitations. As patients received immune modulatory treatment (or not) as part of clinical care or as part of an intervention study rather than in a randomized fashion, and at the same variants of SARS-CoV-2, vaccination rates, and rates of COVID-19 naïve patients changed dramatically over the time course of the study, the results may be biased. For example, patients without any immune modulatory treatment were all admitted before June 2020, while patients receiving tocilizumab were largely included after the initial release of the REMAP-CAP results as a preprint on January 9 2021^52^. Furthermore, if the (non-significant) decrease in mortality in patients treated with tocilizumab combined with corticosteroids compared to corticosteroids alone, is due to treatment alone or (also) due to the aforementioned changes over time, could not be investigated by this study since the number of included patients was too small to answer this question. Second, though we collected comprehensive clinical data and obtained baseline samples as well as follow up samples of all included patients, we relied on established infrastructure for sampling, with slight differences between centers. However, the existing infrastructure is well defined, and within each participating center procedures have been tried and tested for other research questions. Third, the pre-treatment biomarker concentrations showed differences between the groups, suggesting suboptimal balance in unmeasured baseline characteristics. However, since we evaluated the effect of each treatment over time, differences between the biomarker concentrations within the treatment groups could still be assessed reliably. Lastly, as the number of included patients is based on a convenience sample of the three Dutch biobanks that were used, our sample size is relatively small. The results should be interpreted as hypothesis-generating.

## Conclusions

In summary, in this study immunomodulatory treatments for critically ill COVID-19 patients were associated with improvement in biomarkers of dysregulated pathways of complement and endothelial function, potentially suggesting an effect on the stability of the endothelium of these treatments. These results are of interest for potential stratification of patients in further studies on the use of immune modulating treatments in critically ill patients with COVID-19.

### Supplementary Information

Supplementary Information.

## Data Availability

Data can be shared after approval of a proposal with a signed data access agreement and always in collaboration with the study group. For more information, please reach out to the corresponding author (Marleen Slim).
