# Supplementary material for: The effect of immunosuppressive therapies on the endothelial host response in critically ill COVID-19 patients
Source: Sci Rep. 2024 Apr 20;14:9113. doi: 10.1038/s41598-024-59385-w (PMC11032323; doi:10.1038/s41598-024-59385-w)
Supplement: Supplementary file 1 — Supplementary Information. [file 41598_2024_59385_MOESM1_ESM.docx]

**Online Supplement**

**The effect of immunosuppressive therapies on the endothelial host response in critically ill COVID-19 patients**

M.A. Slim, E.H.T. Lim, L.A. van Vught, A.M. Tuip-de Boer, E. Rademaker, J.L.G. Haitsma Mulier, J.J. Engel, the Amsterdam UMC COVID-19 biobank study group, the Radboudumc Center for Infectious Diseases COVID-19 Study Group, P. Pickkers, F.L. van de Veerdonk, A.P.J. Vlaar, L.P.G. Derde, N.P. Juffermans

| **Content** | **Page** |
| --- | --- |
| Supplementary Methods | 2 |
| Supplementary Figure 1. Concentrations of measured biomarkers. | 3 |
| Supplementary Table 1. Biomarkers measured. | 4 |
| Supplementary Table 2. Quality assessment of biomarker measurements. | 5 |
| Supplementary Table 3. Concentrations of biomarkers on baseline in patients with and without pulmonary embolisms. | 6 |
| Supplementary Table 4. Linear mixed-effects models analyses relating concentrations and trends of endothelial host response biomarkers to various immunosuppressive treatments | 7 |

**Supplementary methods**

*Treatment regimens per center*

Patients from the Amsterdam University Medical Centers (Amsterdam UMC) included in this study were treated according to the hospital guidelines at that time. Patients included from the University Medical Center Utrecht (UMCU) who were treated with anakinra, received this treatment as part of the (Randomized Embedded Multifactorial Adaptive Platform Trial for Community Acquired Pneumonia (REMAP-CAP). Patients included from the UMCU who were treated with corticosteroids and/or tocilizumab received this treatment either as part of the REMAP-CAP or later according to the hospital guidelines at that time. Patients from the Radboud University Medical Center Nijmegen (Radboudumc) included in this study were treated with anakinra at the doctor’s discretion.

**Supplementary Figure 1. Concentrations of measured biomarkers.**


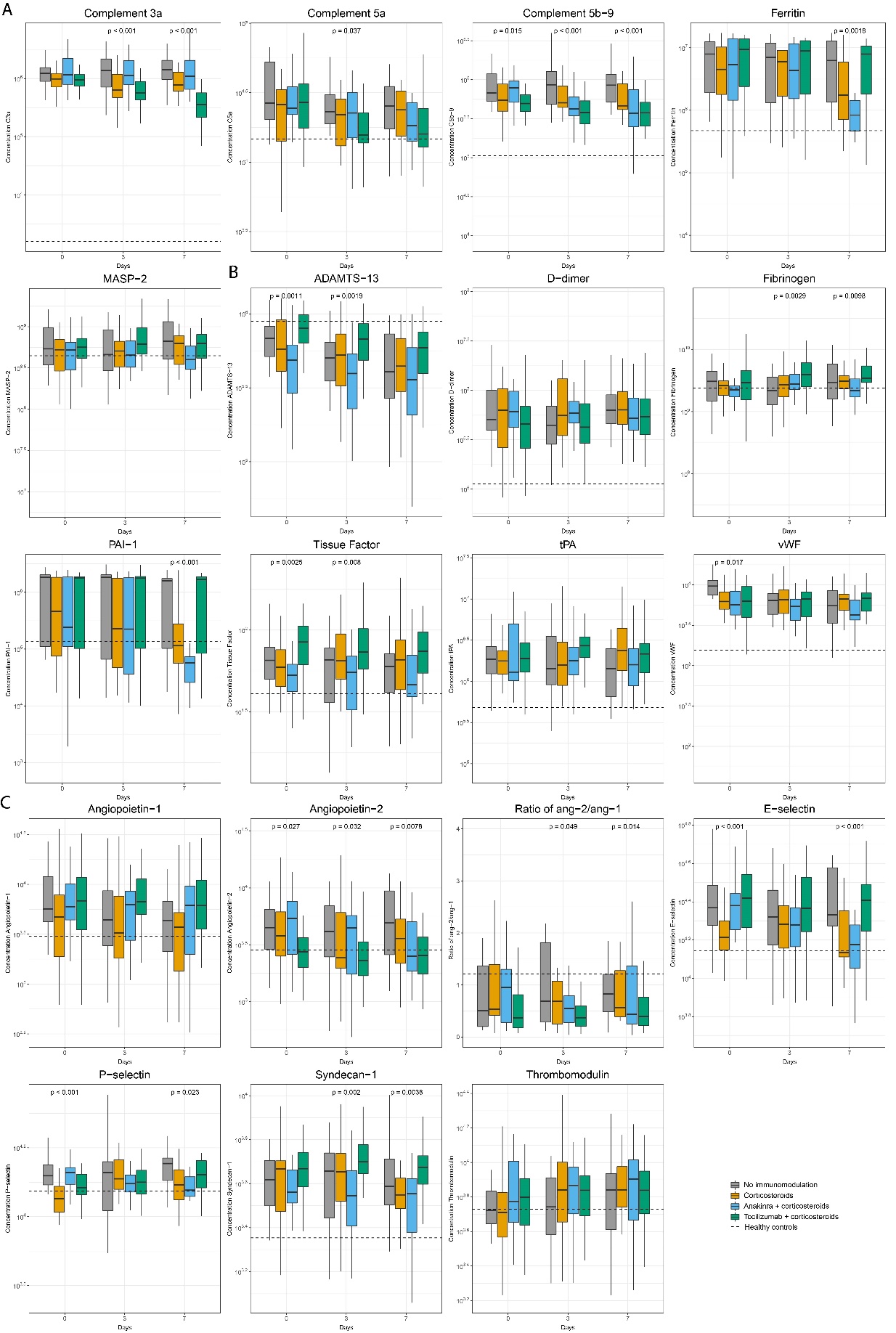


A = Acute phase proteins and complement; B = hemostatis and coagulation, and C = endothelial function. Abbreviations: ADAMTS13, a disintegrin and metalloproteinase with a thrombospondin type 1 motif, member 13; Ang-1, angiopoeitin-1; Ang-2, angiopoeitin-2; C3a, complement 3a; C5a, complement 5a; C5b-9, complement complex 5b-9; MASP-2, Mannan-binding lectin serine protease 2; PAI-1, plasminogen activator inhibitor-1; tPA, tissue Plasminogen Activator; vWF, von Willebrand Factor.

**Supplementary Table 1. Biomarkers measured.**

| **Biomarker** | **Abbreviation** | **Producer** | **Assay** | **Additional information** | **Detection limit* (pg/ml)** |
| --- | --- | --- | --- | --- | --- |
| **Acute phase proteins and complement** | | | | | |
| Complement 3a | C3a | Invitrogen | ELISA | Human C3a ELISA, measured in duplicate | 300-20000 |
| Complement complex 5b-9 | C5b-9 | BD Biosciences |  | Human C5b-9 ELISA , measured in duplicate | 31250-2000000 |
| Mannan-binding lectin serine protease 2 | MASP-2 | Cusabio |  | Human MASP2 ELISA, measured in singular | 23.44-1500 |
| Complement 5a | C5a | R&Dsystems | Luminex | 11-plex Human magnetic luminex assay | 251.3-549580 |
| Ferritin | - |  |  | 2-plex Human magnetic luminex assay | 29341.56-264074.1 |
| **Hemostatis and coagulation** | | | | | |
| Fibrinogen | - | ELabscience | ELISA | Human Fibrinogen ELISA, measured in singular | 15630-1000000 |
| Tissue Plasminogen Activator | tPA | R&Dsystems |  | Human t-Plasminogen Activator ELISA, measured in duplicate | 78.1 – 5.000 |
| Plasminogen activator inhibitor-1 | PAI-1 |  | Luminex | 2-plex Human magnetic luminex assay | 18641.98-167777.8 |
| A disintegrin and metalloproteinase with a thrombospondin type 1 motif, member 13 | ADAMTS-13 |  |  | 11-plex Human magnetic luminex assay | 10611.44-7735800 |
| D-dimer | - |  |  |  | 11741.82-8559800 |
| Tissue Factor | - |  |  |  | 1.04-2260 |
| von Willebrand Factor | vWF |  |  |  | 88.48-21500 |
| **Endothelial function** | | | | | |
| Angiopoeitin-1 | Ang-1 | R&Dsystems | Luminex | 11-plex Human magnetic luminex assay | 56.66-41300 |
| Angiopoeitin-2 | Ang-2 |  |  |  | 18.12-39620 |
| E-selectin | - |  |  |  | 26.66-174980 |
| P-selectin | - |  |  |  | 17.52-114940 |
| Syndecan-1 | - |  |  |  | 26.24-57380 |
| Thrombomodulin | - |  |  |  | 73.16-53340 |

* Detection limit in case of ELISA assays, upper and lower limits of quantification in case of Luminex assays. Abbreviations: ELISA, enzyme-linked immunosorbent assay (ELISA)

**Supplementary Table 2. Quality assessment of biomarker measurements.**

1. **Biomarkers measured with Luminex.**

| **Biomarker** | **Included in analyses*** | **Under LLQ – extrapolated** | **Above ULQ – extrapolated** | **Low acceptable bead count** | **Excluded from analyses**** |
| --- | --- | --- | --- | --- | --- |
| ADAMTS-13 | 99.7% |  |  | 1.7% | 0.3% |
| Ang-1 | 99.4% |  |  | 3.5% | 0.6% |
| Ang-2 | 97.4% | 0.3% |  | 5.8% | 2.6% |
| C5a | 91.9% | 0.9% |  | 25.8% | 8.1% |
| D-dimer | 86.4% |  | 24.3% | 15.9% | 13.6% |
| E-selectin | 98.6% |  |  | 5.2% | 1.4% |
| Ferritin | 98.0% | 1.2% | 45.5% |  | 2.0% |
| PAI-1 | 97.7% | 2.0% |  |  | 2.3% |
| P-selectin | 95.1% | 0.6% |  | 11.6% | 4.9% |
| Syndecan-1 | 99.1% |  |  | 4.6% | 0.9% |
| Thrombomodulin | 96.5% |  |  | 2.0% | 3.5% |
| Tissue Factor | 98.8% |  |  | 2.9% | 1.2% |
| vWF | 86.1% |  |  | 19.6% | 13.9% |

* Included if the following quality assessment was applicable: 1) good beant count or 2) low acceptable bead count AND 1) good quality, 2) under LLQ – extrapolated, or 3) above ULQ – extrapolated. ** Excluded if one of the following quality assessments was applicable: 1) low bead count, 2) under LLQ – set to minimun of standard curve, 3) above ULQ – set to maximum of standard curve, 4) set to non-calculated ULQ, and/or 5) set to non-calculated LLQ. Abbreviations: ADAMTS13, a disintegrin and metalloproteinase with a thrombospondin type 1 motif, member 13; Ang-1, angiopoeitin-1; Ang-2, angiopoeitin-2; C5a, complement 5a; LLQ, lower limit of quantification; PAI-1, plasminogen activator inhibitor-1; vWF, von Willebrand Factor; upper limit of quantification, ULQ.

1. **Biomarkers measured with ELISA.**

| **Biomarker** | **Included in the analyses** | **CV value > 30%*** | **Excluded from analyses**** |
| --- | --- | --- | --- |
| C3a | 92.1% | 12.8% | 7.9% |
| C5b-9 | 98.2% | 0.9% | 1.8% |
| Fibrinogen | 100% | NA*** | 0% |
| MASP-2 | 98.5% | NA*** | 1.5% |
| tPA | 99.7% | 3.4% | 0.3% |

* These measurements are included in the analyses. All analyses were repeated without these measurements, however the results remained the same (data not shown). ** Excluded because values were out of range of the standard curve. *** Fibrinogen and MASP-2 were measured in singular. Abbreviations: C3a, complement 3a; C5b-9, complement complex 5b-9; CV, coefficient of variantion; ELISA, enzyme-linked immunosorbent assay (ELISA)**;** MASP-2, Mannan-binding lectin serine protease 2; tPA, tissue Plasminogen Activator.

**Supplementary Table 3. Concentrations of biomarkers on baseline in patients with and without pulmonary embolisms.**

| **Biomarker** | **Pulmonary embolism**  **(n = 38)** | **No pulmonary embolism**  **(n = 71)** | **p** |
| --- | --- | --- | --- |
| C3a | 1012589.50 [696662.00, 1215973.50] | 1054463.50 [793758.25, 1404703.25] | 0.358 |
| C5a | 29100.40 [18613.20, 38751.04] | 25071.38 [18495.54, 35648.28] | 0.566 |
| C5b-9 | 612402.00 [439301.75, 920552.50] | 555491.00 [438515.75, 714202.00] | 0.593 |
| Ferritin | 9802194.90 [2362635.25, 14292825.50] | 7006640.90 [1592874.77, 12496005.85] | 0.071 |
| MASP-2 | 531343590.00 [362270058.50, 722761999.50] | 548379533.00 [327952905.00, 738765573.00] | 0.720 |
| ADAMTS-13 | 684284.23 (251129.54) | 636066.85 (288374.29) | 0.388 |
| D-dimer | 6260950.00 [3667650.00, 10218925.00] | 4587200.00 [2728100.00, 8291300.00] | 0.128 |
| Fibrinogen | 2579864422.50 [1570335835.00, 3338414965.25] | 2589427797.00 [1943260894.50, 4204486132.00] | 0.445 |
| PAI-1 | 1807948.56 [182226.98, 1918308.86] | 1011184.69 [85602.48, 1859687.01] | 0.049 |
| Tissue Factor | 69.02 [52.08, 81.93] | 60.60 [46.68, 90.01] | 0.731 |
| tPA | 1828537.50 [982610.75, 2572923.00] | 1771919.00 [1064504.00, 2809417.00] | 0.624 |
| vWF | 8023.59 (3635.85) | 7039.67 (3867.07) | 0.230 |
| Ang-1 | 7248.18 [4159.42, 14044.75] | 5479.23 [3381.97, 8529.91] | 0.104 |
| Ang-2 | 3130.76 [2370.22, 5257.33] | 4016.34 [2297.39, 6300.14] | 0.212 |
| Ratio of ang-2/ang-1 | 0.42 [0.21, 1.00] | 0.63 [0.32, 1.62] | 0.101 |
| E-selectin | 19985.04 [16400.99, 29518.58] | 23699.22 [17017.75, 29796.50] | 0.536 |
| P-selectin | 18105.42 [13277.64, 22358.77] | 16865.70 [13883.24, 21875.48] | 0.618 |
| Syndecan-1 | 4103.64 [3274.15, 5139.19] | 4541.04 [3470.76, 5309.10] | 0.378 |
| Thrombomodulin | 5517.29 [4078.30, 7427.36] | 5787.94 [4491.39, 8086.66] | 0.486 |

Data are presented as mean (SD) or median [IQR] when applicable and presented in pg/ml. Abbreviations: ADAMTS13, a disintegrin and metalloproteinase with a thrombospondin type 1 motif, member 13; Ang-1, angiopoeitin-1; Ang-2, angiopoeitin-2; C3a, complement 3a; C5a, complement 5a; C5b-9, complement complex 5b-9; MASP-2, Mannan-binding lectin serine protease 2; PAI-1, plasminogen activator inhibitor-1; tPA, tissue Plasminogen Activator; vWF, von Willebrand Factor.

**Supplementary Table 4. Linear mixed-effects models analyses relating concentrations and trends of endothelial host response biomarkers to various immunosuppressive treatments**

| **A. Treatment with corticosteroids versus no immunomodulation** | | | |  |
| --- | --- | --- | --- | --- |
| **Biomarker** | **Baseline level** | **Time trend per day** | **Effect treatment on level** | **Effect treatment on time trend** |
| C3a | 6.064 (5.938 - 6.189) | 0.011 (-0.010 - 0.033) | -0.188 (-0.360 - -0.015) | 0.016 (-0.047 - 0.015) |
| C5a | 4.446 (4.356 - 4.537) | -0.012 (-0.027 - 0.002) | -0.132 (-0.257 - -0.006) | 0.012 (-0.010 - 0.034) |
| C5b-9 | 5.890 (5.781 - 5.999) | 0.005 (-0.018 - 0.027) | -0.188 (-0.337 - -0.038) | 0.004 (-0.028 - 0.036) |
| Ferritin | 6.603 (6.384 - 6.822) | 0.000 (-0.047 - 0.046) | -0.017 (-0.319 - 0.285) | 0.031 (-0.098 - 0.035) |
| MASP-2 | 8.639 (8.505 - 8.773) | 0.021 (-0.008 - 0.050) | 0.051 (-0.170 - 0.200) | -0.010 (-0.052 - 0.031) |
|  |  |  |  |  |
| ADAMTS-13 | 5.791 (5.703 - 5.879) | -0.027 (-0.039 - -0.015) | -0.064 (-0.183 - 0.055) | 0.016 (-0.001 - 0.034) |
| D-dimer | 6.735 (6.595 - 6.873) | 0.011 (-0.015 - 0.036) | -0.022 (-0.214 - 0.172) | 0.010 (-0.025 - 0.046) |
| Fibrinogen | 9.348 (9.234 - 9.463) | 0.011 (-0.014 - 0.035) | 0.019 (-0.139 - 0.177) | 0.001 (-0.035 - 0.036) |
| PAI-1 | 5.676 (5.375 - 5.977) | -0.010 (-0.070 - 0.050) | -0.118 (-0.527 - 0.290) | -0.043 (-0.129 - 0.041) |
| Tissue Factor | 1.779 (1.693 - 1.865) | -0.008 (-0.021 - 0.005) | 0.029 (-0.088 - 0.147) | 0.008 (-0.012 - 0.027) |
| tPA | 6.262 (6.130 - 6.394) | -0.020 (- -0.048 - 0.008) | -0.066 (-0.248 - 0.116) | 0.041 (0.001 - 0.081) |
| vWF | 3.911 (3.808 - 4.014) | -0.031 (-0.049 - -0.014) | -0.124 (-0.264 - 0.015) | 0.033 (0.008 - 0.058) |
|  |  |  |  |  |
| Ang-1 | 3.832 (3.666 - 3.996) | -0.044 (-0.069 - -0.019) | -0.259 (-0.483 - -0.034) | 0.029 (-0.007 - 0.065) |
| Ang-2 | 3.592 (3.468 - 3.716) | 0.010 (-0.010 - 0.030) | -0.032 (-0.204 - 0.139) | -0.016 (-0.046 - 0.014) |
| Ratio of ang-2/ang-1 | -0.238 (-0.450 - -0.025) | 0.053 (0.025 - 0.081) | 0.216 (-0.080 - 0.512) | -0.043 (-0.085 - -0.001) |
| E-selectin | 4.350 (4.279 - 4.433) | 0.000 (-0.014 - 0.014) | -0.136 (-0.235 - -0.036) | 0.003 (-0.017 - 0.022) |
| P-selectin | 4.257 (4.174 - 4.339) | 0.008 (-0.009 - 0.025) | -0.110 (-0.224 - 0.004) | 0.010 (-0.015 - 0.034) |
| Syndecan-1 | 3.591 (3.523 - 3.658) | 0.001 (-0.012 - 0.014) | 0.021 (-0.073 - 0.114) | -0.007 (-0.025 - 0.012) |
| Thrombomodulin | 3.698 (3.612 - 3.785) | 0.013 (<0.001 - 0.025) | 0.039 (-0.079 - 0.157) | 0.007 (-0.011 - 0.025) |
|  |  |  |  |  |
| **B. Treatment with anakinra and corticosteroids versus no immunomodulation** | | | | |
| **Biomarker** | **Baseline level** | **Time trend per day** | **Effect treatment on level** | **Effect treatment on time trend** |
| C3a | 6.065 (5.934 - 6.195) | 0.012 (-0.009 - 0.032) | -0.021 (-0.208 - 0.166) | -0.018 (-0.049 - 0.015) |
| C5a | 4.445 (4.365 - 4.524) | -0.013 (-0.028 - 0.001) | -0.037 (-0.154 - 0.080) | -0.006 (-0.028 - 0.015) |
| C5b-9 | 5.890 (5.785 - 5.994) | 0.004 (-0.017 - 0.026) | -0.080 (-0.232 - 0.072) | -0.038 (-0.071 - -0.006) |
| Ferritin | 6.559 (6.378 - 6.821) | 0.001 (-0.049 - 0.052) | 0.054 (-0.266 - 0.375) | -0.088 (-0.162 - -0.014) |
| MASP-2 | 8.640 (8.502 - 8.778) | 0.021 (-0.010 - 0.052) | -0.011 (-0.213 - 0.191) | -0.028 (-0.074 - 0.019) |
|  |  |  |  |  |
| ADAMTS-13 | 5.790 (5.696 - 5.882) | -0.027 (-0.041 - -0.014) | -0.175 (-0.308 - -0.041) | 0.014 (-0.005 - 0.034) |
| D-dimer | 6.742 (6.613 - 6.871) | 0.008 (-0.020 - 0.034) | 0.049 (-0.140 - 0.238) | -0.017 (-0.054 - 0.023) |
| Fibrinogen | 9.349 (9.241 - 9.458) | 0.011 (-0.013 - 0.035) | -0.025 (-0.180 - 0.130) | -0.003 (-0.038 - 0.032) |
| PAI-1 | 5.674 (5.368 - 5.979) | -0.008 (-0.072 - 0.055) | -0.096 (-0.527 - 0.334) | 0.094 (-0.186 - -0.002) |
| Tissue Factor | 1.776 (1.696 - 1.856) | -0.009 (-0.022 - 0.004) | -0.066 (-0.180 - 0.049) | 0.004 (-0.016 - 0.023) |
| tPA | 6.263 (6.128 - 6.397) | -0.020 (-0.045 - 0.004) | 0.012 (-0.081 - 0.205) | 0.007 (-0.029 - 0.043) |
| vWF | 3.912 (3.795 - 4.028) | -0.032 (-0.050 - -0.013) | -0.173 (-0.342 - -0.004) | 0.018 (-0.010 - 0.046) |
|  |  |  |  |  |
| Ang-1 | 3.838 (3.686 - 3.991) | -0.043 (-0.064 - -0.022) | -0.091 (-0.310 - 0.128) | 0.033 (0.002 - 0.064) |
| Ang-2 | 3.588 (3.465 - 3.711) | 0.010 (-0.007 - 0.027) | 0.056 (-0.122 - 0.234) | -0.035 (-0.060 - -0.009) |
| Ratio of ang-2/ang-1 | -0.237 (-0.447 - -0.027) | 0.053 (0.029 - 0.077) | 0.138 (-0.164 - 0.439) | -0.071 (-0.107 - -0.034) |
| E-selectin | 4.350 (4.277 - 4.423) | 0.000 (-0.013 - 0.013) | -0.012 (-0.116 - 0.093) | 0.027 (-0.046 - -0.008) |
| P-selectin | 4.256 (4.184 - 4.327) | 0.008 (-0.007 - 0.024) | 0.032 (-0.071 - 0.136) | -0.025 (-0.048 - -0.001) |
| Syndecan-1 | 3.591 (3.527 - 3.654) | 0.000 (-0.013 - 0.013) | -0.014 (-0.105 - 0.077) | -0.013 (-0.032 - 0.005) |
| Thrombomodulin | 3.693 (3.603 - 3.782) | 0.012 (0.000 - 0.024) | 0.116 (-0.013 - 0.245) | -0.010 (-0.028 - 0.008) |
|  |  |  |  |  |
| **C. Treatment with tocilizumab and corticosteroids versus no immunomodulation** | | | | |
| **Biomarker** | **Baseline level** | **Time trend per day** | **Effect treatment on level** | **Effect treatment on time trend** |
| C3a | 6.6067 (5.970 - 6.163) | 0.012 (-0.009 - 0.033) | -0.108 (-0.231 - 0.015) | -0.074 (-0.101 - -0.047) |
| C5a | 4.443 (4.358 - 4.527) | -0.013 (-0.027 - 0.001) | -0.077 (-0.182 - 0.029) | -0.010 (-0.028 - 0.008) |
| C5b-9 | 5.890 (5.805 - 5.976) | 0.004 (-0.014 - 0.022) | -0.210 (-0.319 - -0.102) | -0.023 (-0.047 - 0.000) |
| Ferritin | 6.602 (6.393 - 6.812) | 0.001 (-0.043 - 0.045) | 0.164 (-0.101 - 0.429) | -0.019 (-0.076 - 0.038) |
| MASP-2 | 8.637 (8.507 - 8.767) | 0.021 (-0.005 - 0.047) | 0.120 (-0.045 - 0.0285) | -0.022 (-0.055 - 0.012) |
|  |  |  |  |  |
| ADAMTS-13 | 5.791 (5.712 - 5.869) | -0.028 (-0.040 - -0.015) | 0.042 (-0.056 - 0.140) | 0.013 (-0.004 - 0.029) |
| D-dimer | 6.738 (6.595 - 6.880) | 0.009 (-0.019 - 0.036) | -0.048 (-0.228 - 0.134) | 0.004 (-0.031 - 0.039) |
| Fibrinogen | 9.335 (9.168 - 9.500) | 0.011 (-0.012 - 0.033) | 0.234 (0.029 - 0.443) | 0.016 (-0.013 - 0.046) |
| PAI-1 | 5.674 (5.382 - 5.965) | -0.008 (-0.069 - 0.052) | 0.138 (-0.228 - 0.504) | 0.001 (-0.076 - 0.079) |
| Tissue Factor | 1.776 (1.703 - 1.849) | -0.009 (-0.020 - 0.003) | 0.119 (0.029 - 0.210) | 0.003 (-0.012 - 0.018) |
| tPA | 6.263 (6.127 - 6.399) | -0.021 (-0.43 - 0.002) | -0.013 (-0.184 – 0.157) | 0.031 (0.002 - 0.061) |
| vWF | 3.915 (3.749 - 4.081) | -0.032 (-0.051 - -0.013) | -0.244 (-0.446 - -0.042) | 0.038 (0.013 - 0.063) |
|  |  |  |  |  |
| Ang-1 | 3.838 (3.694 - 3.981) | -0.043 (-0.065 - -0.021) | -0.018 (-0.196 - 0.161) | 0.041 (0.013 - 0.070) |
| Ang-2 | 3.590 (3.480 - 3.700) | 0.010 (-0.008 - 0.027) | -0.143 (-0.280 - -0.005) | -0.021 (-0.043 - 0.002) |
| Ratio of ang-2/ang-1 | -0.238 (-0.415 - -0.060) | 0.053 (0.029 - 0.077) | -0.144 (-0.369 - 0.081) | -0.060 (-0.091 - -0.030) |
| E-selectin | 4.349 (4.272 - 4.426) | 0.000 (-0.013 - 0.013) | 0.059 (-0.037 - 0.156) | -0.006 (-0.022 - 0.011) |
| P-selectin | 4.257 (4.186 - 4.328) | 0.008 (-0.006 - 0.022) | -0.025 (-0.114 - 0.063) | 0.001 (-0.017 - 0.019) |
| Syndecan-1 | 3.591 (3.534 - 3.647) | 0.000 (-0.011 - 0.011) | 0.090 (0.019 - 0.162) | -0.002 (-0.016 - 0.013) |
| Thrombomodulin | 3.694 (3.615 - 3.774) | 0.012 (0.000 - 0.024) | 0.083 (-0.016 - 0.182) | -0.005 (-0.020 - 0.011) |

Data are presented as the interaction term of the model (95% confidence interval) derived from linear mixed-effects model analyses. The treatment group, the sample day, and their interaction were included as fixed effects. Random intercepts were given to each subject. Abbreviations: ADAMTS13, a disintegrin and metalloproteinase with a thrombospondin type 1 motif, member 13; Ang-1, angiopoeitin-1; Ang-2, angiopoeitin-2; C3a, complement 3a; C5a, complement 5a; C5b-9, complement complex 5b-9; MASP-2, Mannan-binding lectin serine protease 2; PAI-1, plasminogen activator inhibitor-1; tPA, tissue Plasminogen Activator; vWF, von Willebrand Factor.
